# *CYP27B1* Gene Polymorphism rs10877012 in Patients Diagnosed with Colorectal Cancer

**DOI:** 10.3390/nu12040998

**Published:** 2020-04-03

**Authors:** Maria Latacz, Jadwiga Snarska, Elżbieta Kostyra, Konrad Wroński, Ewa Fiedorowicz, Huub Savelkoul, Beata Jarmołowska, Janusz Płomiński, Roman Grzybowski, Anna Cieślińska

**Affiliations:** 1 Faculty of Biology and Biotechnology, University of Warmia and Mazury, 10-719 Olsztyn, Poland; mmlatacz@gmail.com (M.L.); ezlbieta.kostyra@uwm.edu.pl (E.K.); ewa.kuzbida@uwm.edu.pl (E.F.); bj58@wp.pl (B.J.); 2 Faculty of Medicine, Collegium Medicum, University of Warmia and Mazury, 10-082 Olsztyn, Poland; 3 Department of General Surgery, Faculty of Medical Sciences, Collegium Medicum, University of Warmia and Mazury, 10-082 Olsztyn, Poland; jadwiga.snarska@uwm.edu.pl; 4 Independent Public Healthcare Center of Ministry of Internal Affairs and Administration with the Warmia and Mazury Center of Oncology, 10-228 Olsztyn, Poland; 5 General and Colorectal Surgery Clinic, University Clinical Hospital of the Military Medical Academy—Central Veterans Hospital, 90-549 Lodz, Poland; konradwronski@wp.pl; 6 Cell Biology and Immunology Group, Department of Animal Sciences, Wageningen University and Research, 6700 AG Wageningen, The Netherlands; huub.savelkoul@wur.nl; 7 Clinical Department of Trauma-Orthopedic Surgery and Spine Surgery of the Provincial Specialist Hospital in Olsztyn, 10-561 Olsztyn, Poland; plominsky@poczta.onet.pl (J.P.); romek.grzybowski@wp.pl (R.G.); 8 Department and Cinic of Orthopaedics and Traumatology, Collegium Medicum, University of Warmia and Mazury, 10-719 Olsztyn, Poland

**Keywords:** rs10877012, colorectal cancer, vitamin D, single nucleotide polymorphism (SNP), CYP27B1

## Abstract

Colorectal cancer (CRC) is the third most commonly occurring cancer worldwide. Intestinal cells are *CYP27B1* gene expression sites and, as a consequence, they are capable of converting pro-vitamin D into the active paracrine and autocrine forms. It was demonstrated that rs10877012 polymorphism in the *CYP27B1* gene influenced the circulating vitamin D level. This provided a rationale for determining the role that this polymorphism plays in the risk of developing colon cancer. In this study, we investigated the association of rs10877012 (T/G) polymorphism in the *CYP27B1* gene with CRC susceptibility. The study population (*n* = 325) included CRC patients (*n* = 106) and healthy controls (*n* = 219). DNA was extracted from peripheral leukocytes and analyzed for the *CYP27B1* polymorphism using the polymerase chain reaction-restriction fragment length polymorphism (PCR-RFLP) method. We found an association between the presence of the T allele at the polymorphic site (odds ratio (OR) = 2.94; 95% CI 1.77–4.86; *p* < 0.0001) and a decreased CRC incidence.

## 1. Introduction

A thorough study of the etiopathogenesis of colorectal cancer (CRC) is one of the major challenges of contemporary medicine. According to Bray et al. [1], there were more than 1.8 million CRC cases in 2018 and nearly 900,000 deaths worldwide. CRC is the third most commonly occurring cancer in mankind, but it is the second most lethal neoplasia [1]. CRC is one of the most common diseases in Poland, and steady increases in morbidity and mortality are being noted simultaneously [2].

**Figure 1:**
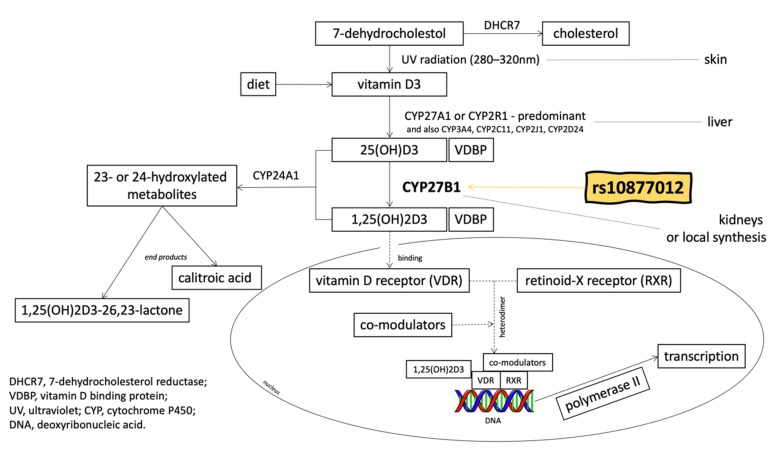
The metabolic pathway of vitamin D (based on Jenkinson 2019 [8] and on Feldman et al. 2014 [9], with modifications).

It is estimated that vitamin D contributes to the expression of 3%–5% of genes, including many related to the development of cancer [3]. By affecting gene expression, vitamin D regulates the following processes: the promotion of apoptosis; induction of cellular differentiation with the simultaneous inhibition of proliferation; inflammation; angiogenesis; invasion; and metastasis. Only the bio-active form of vitamin D, named calcitriol (1,25-dihydroxyvitamin D; 1,25-dihydroxycholecalciferol or 1,25(OH)_2_D_3_), can regulate these gene expressions. Calcitriol is formed by the addition of a hydroxyl group to 25(OH)D as a result of the enzymatic activity of CYP27B1 (Figure 1). Initially, CYP27B1 was only associated with renal tissue (i.e., at the proximal straight tubules and proximal convoluted tubules [4,5]). However, the presence of CYP27B1 has been detected in numerous other cell types, including normal and malignant colon cells [6]. Calcitriol acts by binding to a specific vitamin D receptor (VDR), which is also present in colon cells [7].

Calcitriol plays a role in the regulation of specific signaling pathways in colon cells [9], e.g., it is recognized as a suppressor of the Wnt/ß-catenin signaling pathway (which is important in proliferation) [10]. It is worth mentioning that *APC* (adenomatous polyposis coli) gene mutations (the most common alterations present in colorectal cancer) most presumably lead to an accumulation of ß-catenin [11], and then to an aberration of the WNT signaling pathway. ß-catenin may be one of the important factors in the progression from adenoma to colorectal cancer [12]. SNAIL (zinc finger protein SNAI1) is a protein involved in the process of invasion and metastasis of tumor cells [13]. The expression level of *SNAIL* was inversely correlated with the transcription factor that is associated with vitamin D receptor (*VDR*) expression [14].

The more differentiated the neoplastic cells, the higher the level of *CYP27B1* gene expression that can be observed. This rule even applies to well-differentiated cells found in high-grade (G3) tumors [15]. At the very beginning of the carcinogenesis process, the expression of *CYP27B1* is increased compared to normal, non-pathological mucosa. This increased expression of *CYP27B1* occurs simultaneously with an increased expression of the *VDR*, which potentially affects the anti-proliferative activity of calcitriol in tumor cells [15].

The *CYP27B1* gene is located on 12q14.1 [16] and consists of nine exons [17]. The enzyme is classified in the family of mitochondrial cytochrome P450 enzymes [18]. The translation product is a protein containing 508 amino acids with an N-terminal mitochondrial signal sequence and a heme binding site [18].

The rs10877012 polymorphism is a single nucleotide polymorphism (SNP) located in the promotor region of the *CYP27B1* gene [19]. The reference allele is G, while the alternative allele is T. The minor allele frequency (MAF) oscillates around 27.83% (TopMed study, global population) [19]. Nevertheless, it has been linked directly to CRC occurrence [20,21], and also indirectly due to its influence on the level of 25(OH)D circulating in the blood [22,23]. However, others do not confirm this indirect association [24,25,26]. When it comes to investigating the SNPs in *CYP27B1,* rs10877012 and rs4646536 are the two most frequently chosen variants because of their relatively high MAFs [19]. We decided to choose rs10877012 for three reasons. The first is the detection of its potential role in the development of other malignancies, such as breast cancer [27], hepatocellular carcinoma in patients with hepatitis C virus (HCV) [28] and lung cancer [29]. Secondly, there is no correlation between rs4646536 and CRC [30] or colorectal adenoma [31]. Thirdly, rs10877012 is found in the promotor region and, therefore, its location might impact transcription and translation processes, whereas rs4646536 is an intronic variant [19].

Since the available data do not definitively provide an association between rs10877012 and CRC, further research is indicated. Here, we focused on rs10877012 polymorphism in the *CYP27B1* gene, with reference to susceptibility for the development of colorectal cancer.

## 2. Materials and Methods

Our study involved adult groups, as specified in Table 1; all were members of the Caucasian race. In the study group, there were 106 patients (68 men and 38 women, aged 46–85 years, with a mean age of 64.0 ± 1.8 years) that were diagnosed with CRC and hospitalized between 2011 and 2016 at the Independent Public Health Care Institution of the Ministry of the Interior and Administration, in the Warmian-Masurian Oncology Center in Olsztyn. The healthy control group consisted of 219 healthy people (66 men, 153 women, aged 40–66 years, with a mean age of 54.6 ± 0.7 years).

Adenocarcinoma was diagnosed in all 106 cancer patients, and CRC advancement stages were defined by post-surgical histopathology and clinical evaluation in accordance with TNM staging system. Cancer stage I was determined in 23 patients, stage II in 31 patients, stage III in 29 patients and stage IV in 23 patients.

Both cohorts presented no inflammatory diseases or other infection symptoms, and no urogenital tract or kidney failure. This was confirmed by laboratory tests, and all required data were collected from medical records and/or filled-in questionnaires. All participants gave informed consent for the study, which was performed while respecting ethical standards as well as certified by the Local Bioethics Commission (OIL.492/12/Bioet; 48/2019).

### 2.1. Material Analysis

Approximately 2.0 mL of peripheral blood was collected from 325 participants (106 colorectal cancer and 219 control group) into a tube containing EDTA (ethylenediaminetetraacetic acid).

Genomic DNA was isolated from peripheral blood cells using the GeneJET™ Whole Blood Genomic DNA Purification Mini Kit (Thermo Fisher Scientific, Waltham, MA, USA), according to the manufacturer’s protocol. Polymerase chain reaction-restriction fragment length polymorphism (PCR-RFLP) was performed to genotype the rs10877012 polymorphism. The sequence of the primers examining the polymorphism was as follows: forward primer 5-GCCTGTAGTGCCTTGAGAGG-3, reverse primer 5-CAGTGGGGAATGAGGGAGTA-3 [32].

**Figure 2:**
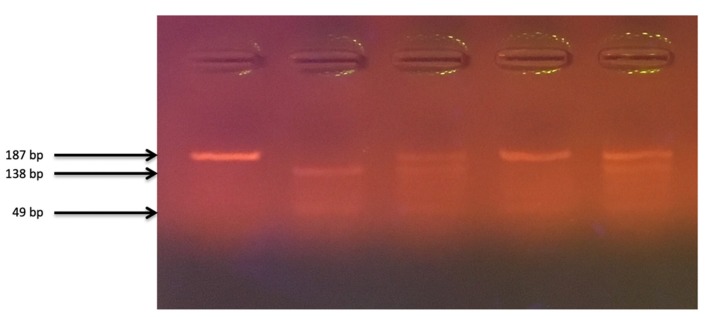
The electropherogram of rs10877012 polymorphism in CYP27B1 gene fragments genotyping. Path 1—PCR product (187 bp); path 2—GG homozygote (138, 49 bp); paths 3 and 5—GT heterozygote (187, 138, 49 bp); path 4—TT homozygote (187 bp).

PCR amplification was conducted in a thermal cycler, according to the following program: initial denaturation: 95 °C for 5 min; proper denaturation: 95 °C for 30 s; attaching the starters at 60 °C for 30 s; synthesis: 72 °C for 60 s; final synthesis: 72 °C for 10 min; number of cycles: 35; cooling: 4 °C. The mixture for amplification in the 18.4 μL volume consisted of DreamTaq™ Green Master Mix (Thermo Fisher Scientific, Waltham, MA, USA); specific primers; the DNA matrix; and ultrapure water (Sigma-Aldrich, Saint Louis, MO, USA). The yield and specificity of the PCR products were evaluated by electrophoresis in 2.5% agarose gel (Promega Corporation, Fitchburg, WI, USA) and staining with GelGreen Nucleic Acid Gel Stain (Biotium, Fremont, CA, USA). Amplified fragments were digested with Fast Digest HinfI G/ANTC restriction enzyme (Thermo Fisher Scientific, Waltham, MA, USA), according to the manufacturer’s instructions, and visualized on a 2.5% agarose gel (Figure 2). Restriction enzyme digestion of the amplicons created fragments of 138 and 49 bp (GG); 187, 138 and 49 bp (GT); and 187 bp (TT). DNA sequencing of random chosen samples after amplification was used to confirm proper genotyping.

### 2.2. Statistical Analysis

The chi-square test was used to analyze the genotype distribution among subjects for the Hardy–Weinberg equilibrium (HWE). Genotypes and SNP allele frequencies were compared in CRC patients to non-CRC control groups by Fisher’s test. Both odds ratios (ORs) and 95% confidence intervals (CIs) were calculated using logistic regression analysis and used to compare both allele frequencies in the study and control cohorts.

The risk of CRC development was estimated via mutant-type allele versus wild-type allele, and via mutant-type genotype versus the wild/mutant and wild-type genotypes. The frequency distribution of age and gender in control and CRC groups was compared using Student’s *t*-test. Statistical analysis was conducted on GraphPad Prism 6 software (GraphPad Software Inc., San Diego, CA, USA), with a *p*-value ≤ 0.05 considered statistically significant.

## 3. Results

The distribution of rs10877012 genotypes were in Hardy–Weinberg equilibrium in both the control group (χ^2^  =  3.138, *p*-value between 0.1 and 0.05) and in the study group (χ^2^  =  0.282, *p*-value between 0.7 and 0.5).

Table 1 presents the characteristics of the study participants. There was a discrepancy between the percentages of men and women in the study group and in the controls. Stage II CRC was found most frequently in the histopathology examination. According to the American Joint Committee on Cancer (AJCC), stage II is characterized by tumor growth into the outermost layers of the colorectal wall (partial or total occupation), or by growth of cancerous cells in the surrounding tissues, but without the presence of metastases to nearby lymph nodes or distant organs. Stage T3 (which is defined as a growth into the outermost layers of the colon or rectum, but without going through them) was additionally the most common among the listed clinical manifestations [33].

Table 2 includes the distribution of both alleles and genotypes at the polymorphic site and associations between the genotype and CRC incidence. Among CRC patients, the G allele was more common (66%), while for healthy controls, its frequency was 53%. As a consequence, the percentage of GG (42%) and GT (47%) was higher in the CRC group. When comparing the alleles TT vs. GT + GG, G was noted to increase the risk of CRC by almost three times, with statistical significance (OR = 2.94; 95% CI 1.77–4.86; *p* < 0.0001). Furthermore, a lower frequency of GT and GG genotypes suggested a decreased CRC incidence.

Table 3 shows the distribution of genotypes by gender. In women compared to men, an additional 2% decrease in the percentage of TT was observed in the CRC group. As in the general population, there was a significantly increased incidence of GG (OR = 4.52; 95% CI 1.26–16.23; *p* = 0.02) and GT (OR = 3.70; 95% CI 1.00–13.66; *p* = 0.049) compared to TT for women diagnosed with CRC. In the male population, the GG genotype appears more frequently (OR = 3.50; 95% CI 1.20–10.17; *p* = 0.02). Due to the already mentioned disproportion in percentages of males and females, there was a lack of statistical relationship between the TT and GT genotypes in men (OR = 1.42; 95% CI 0.53–3.83; *p* = 0.49).

## 4. Discussion

The increased colorectal cancer incidence and number of deaths create an urgent need to identify numerous factors that could play a role in its etiopathogenesis. After the demonstration that intestinal cells are capable of converting vitamin D into active paracrine and autocrine forms [6], the influence of *CYP27B1* polymorphism on vitamin D has become a potential subject of research for determining the risk of colon cancer occurrence. Moreover, a few papers have shown that genetic variants at the rs10877012 polymorphic site in *CYP27B1* affect the serum vitamin D level [22,23,34,35], but still, the question remains as to how the rs10877012 polymorphism influences intestinal carcinogenesis.

The results presented in Table 2 suggest a significant effect of rs10877012 polymorphism on the occurrence of CRC. Our data suggest a protective role of the T allele (for TT vs. GT + GG: OR = 2.94; 95% CI 1.77–4.86; *p* < 0.0001) and the TT genotype (for GT vs. TT: OR = 2.56; 95% CI 1.23–5.32; *p* = 0.01; for GG vs. TT: OR = 3.20; 95% CI 1.51–6.77; *p* = 0.002). Contrary to our findings, Vidigal et al. (whose study was based in Brazil) indicate that in the male population the presence of T allele increases the risk of CRC by 1.60-fold (*p* = 0.037), while the presence of GT genotype—by 2.04-fold (*p* = 0.014) [20]. The MAF in Latin Americans is similar to the MAF in Europeans, but the difference could cause the discrepancies in results between the papers [19]. For males and females, the association of GT+TT vs. GG caused a 2.14-fold greater risk for neoplasia (*p* = 0.003) [20]. Comparison of GG vs. TT in the whole population: we obtained a 3.20-times (*p* = 0.002) higher risk of CRC, whereas for Vidigal et al.’s population the result was not statistically significant (*p* = 0.412) [20]. Another paper demonstrated that the GG genotype, compared to the TT genotype, diminished the risk of CRC (OR = 0.57; 95% CI 0.38–0.84; *p* = 0.005), which turned out to be only statistically significant in elderly people [21]. It is worth mentioning that this latter study was performed in a population of northeast China, the wild-type genotype was TT where (in our study it was GG) [21]. To our knowledge, the two citied papers [20,21] are the only references in the PubMed database that directly associate rs10877012 to the development of CRC.

The polymorphisms in *CYP27B1* that have been linked to the occurrence of CRC are: rs28934604, rs58915677, rs13377933 and rs2229103 [36], as well as rs4646536 and rs10877013 [37]. The number of published papers reflecting SNPs in *CYP27B1* is also limited; most information available relates to single nucleotide variations in *VDR* (research articles, meta-analysis, and even randomized clinical trials) [20,37,38,39].

It is worth mentioning that numerous studies link the rs10877012 polymorphism to 25(OH)D levels [22,23,34,35]. The plasma level of 25(OH)D has been established as an indicator of the vitamin D status in the organism [10]. It is suggested that higher levels of vitamin D are negatively correlated with the risk of colorectal neoplasia [40]. Based on these data, an assumption can be made that papers confirming an association between the polymorphism and the level of 25(OH)D indirectly confirm a protective role of vitamin D in carcinogenesis.

Our study did not include actual measurement of serum vitamin D levels because the blood samples were collected at different times of the year in both cohorts; concentration measurements would therefore be meaningless. While the season of the year is associated with the vitamin D status, Bu et al. indicated that age and gender did not influence the level of 25(OH)D [24]. Moreover, diet and exposure to sunlight are two additional factors that contribute to the plasma levels of this molecule to the greatest extent in the human body [41]. Supplementation is an easy method of monitoring the total vitamin D intake, while the precise control of dietary intake of vitamin D content is rather difficult outside of a hospital environment, not to mention the sunlight exposure control. Last but not least, colorectal carcinogenesis is a multifactorial process and, apart from vitamin D, also a low intake of folic acid, fiber, calcium, high red meat consumption [42] and aberrations in proper functioning of the gut microbiota [43] are relevant influencing factors.

It has been established that cytochrome P450 (CYP) enzymes are vital to the process of carcinogenesis [44]. Their action in the oxidation of substrates can cause formation of carcinogens, but on the other hand, such a reaction also provides a substrate for phase II of the metabolism of xenobiotics, and the chemical products of this reaction (polar and mostly not harmful) can be excreted via the kidneys [44]. In addition, CYPs are also responsible for bioactivation or deactivation of chemotherapeutic agents [45].

The mutations in these *CYP* genes will have different influences on the activity of the gene products [46]. Based on existing phenotypes, Ingelman-Sundberg divided humans into four groups: poor metabolizers (lacking functional enzymes), intermediate metabolizers (heterozygotes with one defective allele), efficient metabolizers (two functional gene copies) and ultrarapid metabolizers (with more than two functional gene copies) [47]. As already mentioned, the main action of CYP27B1 is the formation of the bio-active form of vitamin D from 25(OH)D. Because the impact of rs10877012 on *CYP27B1* expression is not fully known, the measurement of 25(OH)D levels is only a downstream effect of the potential impact. Generally, 25(OH)D plasma levels are low in colorectal cancer patients [48], while the detection of bio-active 1,25(OH)_2_D is hard to perform due to the short half-life of this molecule (i.e. 4–6 h) [49], and because intestinal cells synthesize calcitriol in an autocrine or paracrine manner, without releasing it into the bloodstream [6].

In conclusion, genotyping is an easy and robust procedure that has the additional advantage of having to be performed only once. Creating a panel of relevant SNPs associated with CRC may contribute to the early detection and possible prevention of the illness owing to the identification of predisposing genotypes.

## Figures and Tables

**Table 1:** Clinical characteristics of patients and control group. Bracket numbers indicate the percentage of patients relative to the total study group.

	CRC Patients Group (*n* = 106)	Control Group (*n* = 219)	*p*-Value
Age: *years (mean ± SEM)*	64.0 ± 1.8	54.6 ± 0.7	<0.0001
Gender, *n* (%)			
Female	38 (35.8)	153 (69.8)	
Male	68 (64.1)	66 (30.1)	<0.0001
Tumor stage, *n* (%)			
I	23 (21.7)		
II	31 (29.2)		
III	29 (27.4)		
IV	23 (21.7)		
Pathological tumor stage (pT), *n* (%)			
T1	0 (0)		
T2	22 (20.8)		
T3	64 (60.4)		
T4	20 (18.8)		
Pathological nodal status (pN), *n* (%)			
no lymph node metastasis	50 (47.2)		
lymph node metastasis	56 (52.8)		
Pathological metastasis status (pM), *n* (%)			
no distant metastasis	66 (62.3)		
metastasis to distant organs	40 (37.7)		

Abbreviations: CRC—colorectal cancer; SEM—standard error of the mean.

**Table 2:** Genotype and allele frequencies of *CYP27B1* single nucleotide polymorphism (SNP) rs10877012 polymorphism in CRC and control groups.

Genotype/Allele	CRC *n* (%)	Control *n* (%)	OR (95% CI) Control vs. CRC	*p*-Value
TT	11 (10)	54 (25)	-	-
GT	50 (47)	96 (44)	2.56 (1.23–5.32)	0.01
GG	45 (42)	69 (32)	3.20 (1.51–6.77)	0.002
G	140 (66)	234 (53)		
T	72 (34)	204 (47)		
**Control vs. CRC**				
**TT vs.**	22 (14)	108 (32)		
**GT + GG**	140 (86)	234 (68)	2.94 (1.77–4.86)	<0.0001

Genotype frequencies of *CYP27B1* SNP were determined in CRC patients and in the non-CRC control group. ORs with 95% CI and *p*-values were calculated for the TT genotype versus the GT and GG genotypes.

**Table 3:** Genotype and allele frequencies of *CYP27B1* SNP rs10877012 polymorphism in the studied groups.

**For Females**				
**Genotype/Allele**	**CRC** ***n* (%)**	**Control** ***n* (%)**	**OR** **(95% CI)** **Control vs. CRC**	*****p***** **-Value**
TT	3 (8)	40 (26)	-	-
GT	20 (53)	59 (39)	4.52 (1.26–16.23)	0.02
GG	15 (39)	54 (35)	3.70 (1.00–13.66)	0.049
**Control vs. CRC**				
**TT vs.**	6 (11)	80 (32)		
**GT + GG**	50 (89)	167 (68)	3.99 (1.64–9.70)	0.0022
**For Males**				
**Genotype/Allele**	**CRC** ***n* (%)**	**Control** ***n* (%)**	**OR** **(95% CI)** **Control vs. CRC**	*****p***** **-Value**
TT	8 (12)	14 (21)	-	-
GT	30 (44)	37 (56)	1.42 (0.53–3.83)	0.49
GG	30 (44)	15 (23)	3.50 (1.20–10.17)	0.02
**Control vs. CRC**				
**TT vs.**	16 (15)	28 (29)		
**GT + GG**	90 (85)	67 (71)	1.31 (0.64–2.67)	0.46

Genotype frequencies of *CYP27B1* SNP were determined in CRC patients and in the non-CRC control group. ORs with 95% CI and *p*-values were calculated for the TT genotype versus the GT and GG genotypes.
